# Comprehensive pregnancy monitoring with a network of wireless, soft, and flexible sensors in high- and low-resource health settings

**DOI:** 10.1073/pnas.2100466118

**Published:** 2021-05-10

**Authors:** Dennis Ryu, Dong Hyun Kim, Joan T. Price, Jong Yoon Lee, Ha Uk Chung, Emily Allen, Jessica R. Walter, Hyoyoung Jeong, Jingyue Cao, Elena Kulikova, Hajar Abu-Zayed, Rachel Lee, Knute L. Martell, Michael Zhang, Brianna R. Kampmeier, Marc Hill, JooHee Lee, Edward Kim, Yerim Park, Hokyung Jang, Hany Arafa, Claire Liu, Maureen Chisembele, Bellington Vwalika, Ntazana Sindano, M. Bridget Spelke, Amy S. Paller, Ashish Premkumar, William A. Grobman, Jeffrey S. A. Stringer, John A. Rogers, Shuai Xu

**Affiliations:** ^a^Sibel Inc., Niles, IL 60714;; ^b^Department of Obstetrics and Gynecology, University of North Carolina at Chapel Hill, Chapel Hill, NC 27599;; ^c^University of North Carolina Global Projects–Zambia, Lusaka 10101, Zambia;; ^d^Querrey Simpson Institute for Bioelectronics, Northwestern University, Evanston, IL 60208;; ^e^Department of Electrical and Computer Engineering, Northwestern University, Evanston, IL 60208;; ^f^Department of Obstetrics and Gynecology, Northwestern University Feinberg School of Medicine, Chicago, IL 60611;; ^g^Department of Materials Science and Engineering, Northwestern University, Evanston, IL 60208;; ^h^Department of Biomedical Engineering, Northwestern University, Evanston, IL 60208;; ^i^Department of Obstetrics and Gynecology, University of Zambia School of Medicine, Lusaka 10101, Zambia;; ^j^Women and Newborn Hospital, University Teaching Hospital, Lusaka 10101, Zambia;; ^k^Department of Dermatology, Northwestern University Feinberg School of Medicine, Chicago, IL 60611;; ^l^John H. Stroger, Jr. Hospital of Cook County, Chicago, IL 60612;; ^m^Department of Anthropology, The Graduate School, Northwestern University, Evanston, IL 60208;; ^n^Department of Neurological Surgery, Feinberg School of Medicine, Northwestern University, Chicago, IL 60611

**Keywords:** biosensors, pregnancy, vital signs

## Abstract

Vital signs monitoring is a fundamental component of ensuring the health and safety of women and newborns during pregnancy, labor, and childbirth. This monitoring is often the first step in early detection of pregnancy abnormalities, providing an opportunity for prompt, effective intervention to prevent maternal and neonatal morbidity and mortality. Contemporary pregnancy monitoring systems require numerous devices wired to large base units; at least five separate devices with distinct user interfaces are commonly used to detect uterine contractility, maternal blood oxygenation, temperature, heart rate, blood pressure, and fetal heart rate. Current monitoring technologies are expensive and complex with implementation challenges in low-resource settings where maternal morbidity and mortality is the greatest. We present an integrated monitoring platform leveraging advanced flexible electronics, wireless connectivity, and compatibility with a wide range of low-cost mobile devices. Three flexible, soft, and low-profile sensors offer comprehensive vital signs monitoring for both women and fetuses with time-synchronized operation, including advanced parameters such as continuous cuffless blood pressure, electrohysterography-derived uterine monitoring, and automated body position classification. Successful field trials of pregnant women between 25 and 41 wk of gestation in both high-resource settings (*n* = 91) and low-resource settings (*n* = 485) demonstrate the system’s performance, usability, and safety.

## Significance

Monitoring vital signs for laboring women and their fetuses is foundational to the delivery of obstetrical care; however, monitoring platforms for pregnancy have undergone little innovation over the last several decades with many low-income settings lacking basic access. We report a new time-synchronized, flexible, and wireless sensor system applicable across the entire continuum of antepartum and postpartum care that provides continuous, comprehensive, and noninvasive monitoring (heart rate, respiratory rate, and pulse oxygenation) compatible with a wide range of mobile devices. This system offers advanced features such as continuous blood pressure, uterine electrohysterography, and automated body position classification. We further demonstrate the performance of this new system among pregnant individuals (*n* = 576) in both high-resource settings and low-resource care settings.

Pregnancy is experienced by more than 200 million people annually (1). Although pregnancy, labor, and delivery are common events, there remains a continued risk of catastrophic outcomes, with 23.8 maternal deaths per 100,000 births in the United States and 462 deaths per 100,000 births in low-income countries (2). Accordingly, ∼295,000 women die as a result of childbirth complications each year, with 94% of these deaths occurring in low- and middle-income countries (LMICs) (3). The most common causes of maternal morbidity and mortality—hemorrhage, hypertensive disorders, infection, and sepsis—are often preceded by predictable vital signs perturbations in both high-resource and low-resource settings, and early intervention is key to improving outcomes (4–9).

Standard monitoring systems have changed minimally over the past 30 y (10). The most common systems for monitoring fetal heart rate (FHR) and uterine contractions during labor are rigid tocodynamometer systems (each typically 7- to 9-cm diameter in size) affixed to a patient’s abdomen with straps that have accompanying wires tethered to a wall-mounted base unit. A Doppler sensor measures FHR and a tocodynamometer measures uterine contraction. This limits a person’s mobility and comfort during labor. Unsurprisingly, pregnancy monitoring with cardiotocography (CTG) is therefore largely limited to intrapartum monitoring, facilitated by a hospital’s infrastructure. Additional monitoring equipment designed to assess maternal or fetal status, such as pulse oximetry, blood pressure (BP) monitors, and electrocardiography (ECG), are typically used in a noncontinuous manner and place a relatively high burden on providers (11). The failure to recognize and act on deteriorating vital signs is identified as a key contributor to preventable maternal deaths (12, 13). Cumulatively, the high base costs, expensive consumables, and operational burden of existing monitoring systems often preclude deployment in LMIC settings where maternal morbidity is the highest. In many parts of the world, a partograph, a simple graphical representation of maternal and fetal progress through labor, remains the only available method used to track labor despite a lack of evidence of clinical benefit for laboring women (14). In more developed countries, commercial innovation and growth have produced multiple home pregnancy monitoring systems. The Invu (formerly PregSense) from Nuvo Inc. and Bloomlife Pregnancy Tracker from Bloomlife Inc. aspire to bring the same care and attention available in the labor ward to the home. Such platforms, however, are not sufficiently comprehensive to replicate full clinical monitoring. The Invu reports both FHR and maternal HR but does not capture uterine contractions (15). The Bloomlife Pregnancy Tracker captures electrohysterography (EHG) without fetal ECG (16). Other systems, such as the Novii Wireless Patch System from Monica Healthcare, are available to acquire fetal ECG, but such systems alone cannot monitor nonsingleton pregnancies and are limited to term pregnancies (at least 37 wk of gestation) (17). While these devices excel in capitalizing on fetal measurements, they often overlook the importance of measuring core maternal vital signs of laboring women. Beyond these specific efforts, broader advances in both wearables, flexible electronics, and analytics have demonstrated the ability to monitor both traditional vital signs and novel digital biomarkers which hold future relevance for women’s health (18–23). Herein, we present a comprehensive system able to monitor all core vital signs for both the fetus and mother in the form of soft, flexible electronics with advanced analytics and wireless operation validated for performance in both low-and high-resource healthcare settings.

There is a critical need for new classes of advanced maternal monitoring systems that offer comprehensive and continuous clinical-grade vital signs measurements, advanced monitoring capabilities to support clinical decision making, and operability in both high-resource and low-resource settings. We present an integrated monitoring platform leveraging advanced soft electronics, wireless connectivity, and compatibility with a wide range of low-cost mobile devices. This platform contains three flexible, soft, and low-profile sensors that offer comprehensive vital signs monitoring for both a woman and a fetus with time synchronized operation, allowing for advanced parameter assessment, such as continuous cuffless BP, EHG-derived uterine monitoring, and automated body position classification. The data streams generated by this system are integrated with the cloud, thereby allowing for future machine-learning opportunities to predict maternal or neonatal deterioration. Successful field trials among pregnant women between 25 and 41 wk of gestation (*n* = 576) in both high-resource (Chicago, IL) and low-resource (Lusaka, Zambia) settings for pregnant women demonstrate the system’s performance and usability.

## Results

### Wireless Maternal–Fetal Sensor System Design.

**Fig. 1.**
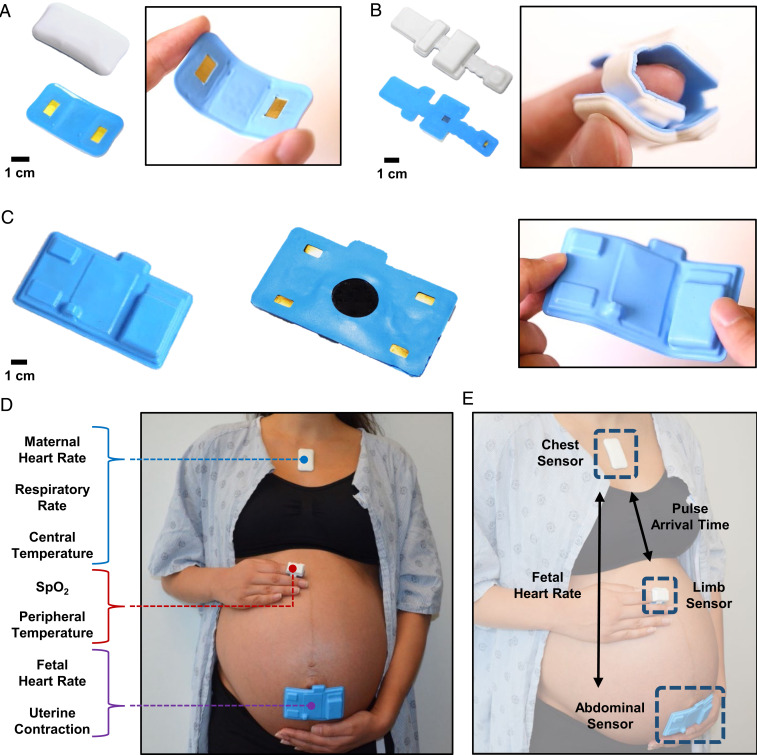
Overview of the maternal fetal monitoring system. The on-body network of the maternal–fetal sensor system is visualized. (*A* and *B*) Photographs of the front and back side of the chest and limb sensors. The silicone encapsulation allows the sensors to be soft and stretchable while still operating wirelessly. (*C*) The abdominal sensor is designed to conform around the abdomen of the patient without the need for an external strap. (*D*) Individually, each sensor captures unique signals from the patient. (*E*) Together, the sensors constitute an on-body network capable of acquiring more advanced metrics.

Fig. 1 *A*−*C* provide a front and back view of the chest, limb, and abdominal sensors, respectively. The chest sensor (Fig. 1*A*) measures ECG, seismocardiogram (SCG), chest wall movement for respiratory rate (RR), and skin temperature, each sampled at 512, 416, 52, and 0.2 Hz, respectively. The chest sensor includes a biopotential analog front end (AFE) (MAX30001; Maxim Integrated), a high-frequency three-axis inertial measurement unit (IMU) (LSM6DSL; STMicroelectronics), and a clinical-grade thermometer (MAX30205; Maxim Integrated). The biopotential AFE measures ECG by passively reading the electrical impulses of the heart. The IMU uses the three-axis accelerometer to measure chest-wall movement. The clinical-grade thermometer measures surface temperature through contact sensing on the underside of the chip. Once packaged, the chest sensor is placed at an oblique angle on the chest of the participant, starting from the midline of the body and following the collarbone upward. We demonstrate the form factor of the chest sensor, measuring 4.8 cm in length, 3.4 cm in width, and 11.7 g in weight. The incorporation of serpentine lines and subislands in the flexible printed circuit board allow the sensor to be stretched and bent as shown in Fig. 1*A*. The underside of the chest sensor exposes the two gold electrodes, which face the skin. The limb sensor (Fig. 1*B*) measures photoplethysmogram (PPG) and skin temperature, sampled at 256 and 0.2 Hz, respectively. The limb sensor wraps around the index finger of the participant to collect PPG and peripheral skin temperature. The limb sensor is composed of an integrated pulse oximetry module (MAX86141; Maxim Integrated) and a clinical-grade thermometer (MAX30205; Maxim Integrated). The integrated optical AFE controls the red and infrared light-emitting diodes and photodiode to acquire the PPG signal from under the skin, which is used to measure peripheral capillary oxygen saturation (SpO_2_). The same clinical-grade thermometer measures skin temperature. We demonstrate the form factor of the limb sensor, measuring 9.1 cm in length, 3.1 cm in width, and 9.5 g in weight. Unlike the chest senor, which is placed flat on the skin, the limb sensor wraps circumferentially around the finger of the user as shown in Fig. 1*B*, emphasizing the elastic and mechanical properties of the sensor. The abdominal sensor (Fig. 1*C*) offers an onboard wireless Doppler ultrasound (US), EHG, and ECG, each sampled at 504 and 500 Hz, respectively. This sensor is placed on the participant’s abdomen to derive FHR via US, EHG to derive uterine contraction via EHG, and maternal HR via ECG. The EHG and ECG functionalities are acquired by a low-noise biopotential unit (ADS1299-4; Texas Instruments), A total of four electrodes are located on the lateral aspects of the abdominal sensor: two for channel readings, one for reference measurement, and one for bias measurement. The form factor of the abdominal sensor is shown, measuring 10 cm in length, 5.7 cm in width, and 37.3 g in weight. Because the abdominal sensor must be placed on a curved surface, we demonstrate the ability of the sensor to bend and conform to the curvature of a gravid abdomen in Fig. 1*C*. The underside of the abdominal sensor exposes four gold electrodes and the ultrasonic transducer, which face the skin. The chest and limb sensors have an operation time of 24 h and the abdominal sensor has an operation time of 8 h. The chest, limb, and abdominal sensors all integrate commercially available components to not only take advantage of state-of-the-art technology but also to facilitate manufacturing for mass production.

Fig. 1*D* demonstrates the comprehensive maternal–fetal monitoring setup on a gravid patient. The chest sensor is placed under the suprasternal notch, the limb sensor is wrapped around the index finger, and the abdominal sensor is placed just below the umbilicus. The chest sensor measures maternal HR, RR, and central temperature from the patient. The limb sensor measures oxygen saturation and peripheral temperature. The abdominal sensor measures FHR and uterine contraction from the patient. All three sensors connect to a central mobile tablet which serves as the base station for Bluetooth Low Energy communication and real-time data display. A biocompatible, double-sided hydrogel adhesive conducts the signal from the patient to the chest and abdominal sensors and its adhesion strength has been deemed safe to use by a board-certified dermatologist. The adhesive is placed on the exposed gold leads of the sensors. The sensor is able to obtain high signal quality even in the presence of motion and is water-resistant such that it can be worn in the shower. A latex-free, cotton fabric strap holds the limb sensor in place around the index finger. Standard US gel is used on the central component of the abdominal sensor for fetal Doppler. Fig. 1*E* demonstrates the on-body network achieved via out-of-band time synchronization of the three sensors with 1-ms timing accuracy. The time synchronization infrastructure was previously established and yields an SD of 3.6 ms over 24 consecutive hours (22). The chest sensor acts as the central node, with the abdominal and limb unit sensors acting as peripherals. The outputs of the chest and limb sensors are then capable of calculating pulse arrival time (PAT), which serves as a surrogate measurement for BP (24, 25). The chest and abdominal sensors also communicate, which enables maternal ECG elimination from the abdominal sensor’s outputs, allowing for fetal ECG isolation. Real-time comparison of maternal HR and FHR is also displayed on the software interface. *SI Appendix*, Fig. S1 outlines a block diagram illustrating the high-level architecture of the system for the chest, limb, and abdominal sensors. The methods provide detailed information on the sensors’ construction.

### Real-Time Measurement of Clinical Data for Maternal Health.

**Fig. 2.**
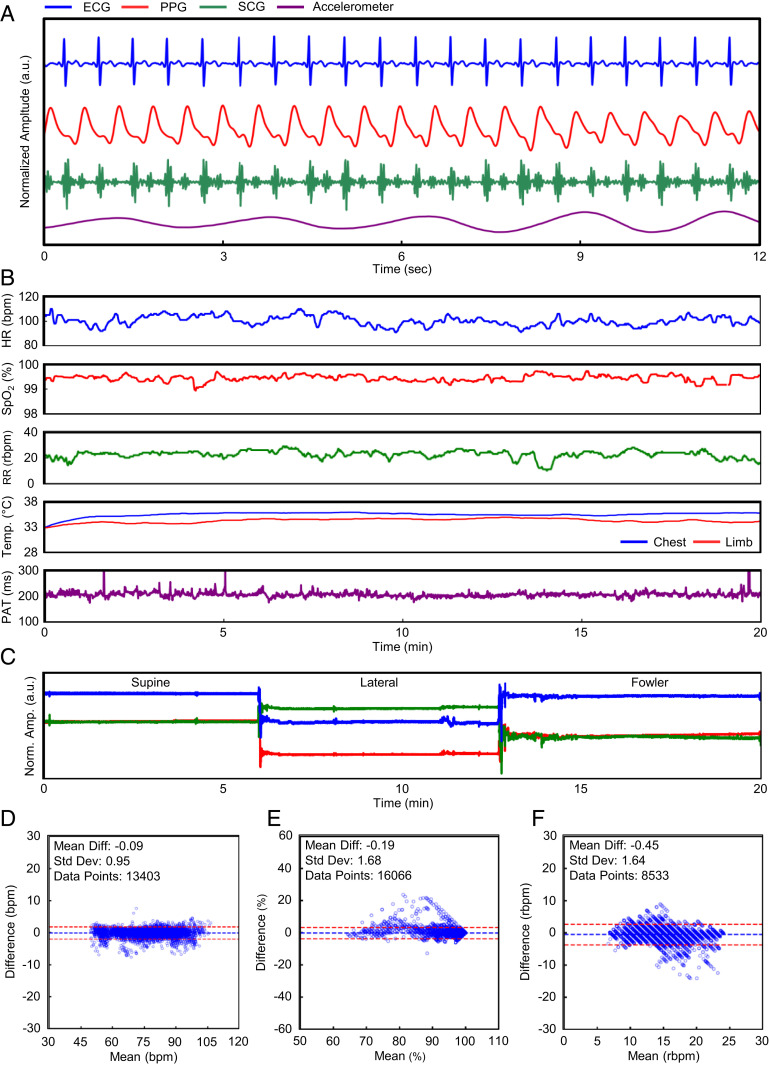
Maternal vital signs. The end-to-end data analytics of the sensor system is outlined. (*A*) Waveforms from the patient are obtained by the chest, limb, and abdominal sensors. (*B*) The raw signals are then processed to yield the representative vital signs of a clinical setting. (*C*) The patient’s body orientation is also identifiable through the use of an embedded IMU. (*D–F*) Our calculated metrics are statistically comparable to the gold standard used in modern hospitals.

Fig. 2*A* summarizes the core sensor outputs of the wireless sensor network for maternal ECG, PPG, SCG, and three-axis accelerometry. The real-time data are processed via algorithms implemented directly on a mobile device to calculate vital signs. Fig. 2*B* depicts vital signs processed in real time from the sensor system in a time-series format. A modified Pan–Tompkins algorithm filters the data and R-peak detection yields maternal HR. SpO_2_ is calculated by filtering the red and infrared channels, detecting peaks, and calculating the ratio of pulse amplitudes. RR is captured by fusion chest wall movement data obtained from the *x* and *y* axis of the accelerometer and the ECG signal. Finally, continuous temperature measurements on the chest and limb sensors obtained from direct readings allow for fever curve tracking. Temperature measurements may also be used as an indication of peripheral perfusion and volume status in the setting of shock, which are particularly relevant in the context of sudden blood loss related to an obstetrical emergency (26–28). Fig. 2*C* demonstrates the classification of accelerometer movement into body postures. Different baseline levels obtained from the *x*, *y*, and *z* axes correspond to differing orientations of the chest sensor on the participant. After classifying the different states of body position, we can compare the correlation between posture and vital signs. In Fig. 2 *D*–*F* we demonstrate the agreement in HR, SpO_2_, and RR calculations between the experimental system and a commercially available gold-standard monitoring system (Intellivue MP50; Philips) operating concomitantly in a cohort of 13 healthy nonpregnant adults. The Bland–Altman method provides a quantitative comparison method and supports similarity between our sensors and the gold-standard monitoring system. The mean difference is −0.09 beats per minute (bpm) for HR (SD 0.95 bpm), −0.19% for SpO_2_ (SD 1.68%), and −0.45 breaths per minute for RR (SD 1.64 breaths per minute). The differences are within Food and Drug Administration guidelines for new cardiopulmonary monitoring platforms (29, 30). Data can be streamed continuously or hidden for clinical research purposes using a wide range of mobile devices (*SI Appendix*, Fig. S2).

### FHR and Uterine Contraction via a Soft, Flexible, and Wireless Abdominal Sensor.

**Fig. 3.**
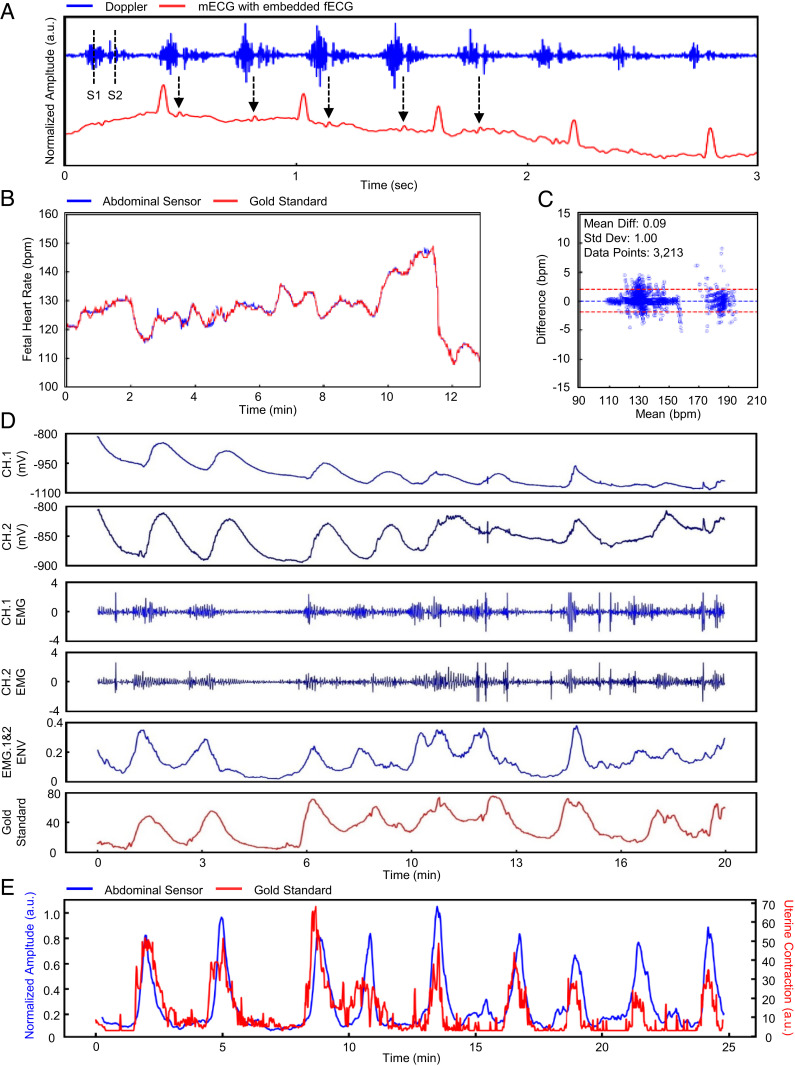
Doppler-derived FHR and EMG-derived uterine contraction. Data analytics of the FHR and maternal uterine contraction are outlined. (*A*) The raw US Doppler signal is obtained by the abdominal sensor. We can identify the S1 and S2 waves and see the signal aligned with peaks indicative of fetal ECG. (*B* and *C*) Our calculated FHR is statistically comparable to the gold standard. (*D*) The raw biosignal is obtained by the abdominal sensor. We acquire two channels for sequential processing of the EMG signal. (*E*) Our calculated uterine contraction output is overlaid onto the gold standard.

Continuous FHR is commonly tracked in high-resource settings during labor via wired Doppler US pucks strapped to the abdomen. The wireless abdominal sensor presented here generates a US waveform at a set frequency via an onboard transducer. Fig. 3*A* illustrates the raw Doppler signal collected from the abdominal sensor. There are two distinct peaks identifiable in the signal. The first peak “S1” represents closure of the tricuspid and mitral valves of the fetal heart, and the second peak “S2” represents closure of the pulmonary semilunar valves of the fetal heart (Audio S1). A sequence of the two peaks constitutes one fetal heartbeat. To replicate the audible Doppler signal available in a gold-standard tocodynamometer (GE Corometrics 250cx; GE Healthcare), we normalized the signal to a scale between [−1, 1] and encoded to the original sampling rate of 504 Hz. The result is an audio file which produces an audio output of fetal Doppler. Although intended for measuring maternal ECG, maternal HR, and uterine contraction, the abdominal sensor is also capable of capturing incidental fetal ECG, which coincides with fetal Doppler signal. Larger peaks can be identified as maternal ECG, and smaller peaks are indicative of the fetal ECG, typically an order of magnitude lower in amplitude. In a subset of people the abdominal sensor output was analyzed to isolate fetal ECG; the R-to-R peak interval for maternal ECG averaged 635 ms, and the fetal ECG R-to-R peak interval averaged 344 ms. The R-to-R peak intervals translate to 94 bpm and 170 bpm for maternal and fetal HR, respectively. Further development efforts are targeting enhancement of fetal ECG capabilities of this system. A flow diagram of our algorithm to derive FHR via wireless US is outlined (*SI Appendix*, Fig. S3*A*). Fig. 3*B* illustrates FHR calculation between our abdominal sensor and gold-standard Doppler US systems. The sensor tracks expected FHR variation over time. A Bland–Altman plot comparing data from the abdominal sensor to the gold standard is presented in Fig. 3*C* for a subset of *n* = 10 laboring women who contributed 3,213 single data points. The mean difference is 0.1 bpm with an SD of 1 bpm, showing strong agreement.

In Fig. 3*D* we illustrate the derivation of uterine contraction from the abdominal sensor’s EHG capabilities. The contraction of the uterine muscle is sensed at the skin surface. The EHG contraction can be converted to millimeters of mercury (mmHg) or pressure with a simple calibration. Fig. 3*E* illustrates a time-series comparison of EHG-derived uterine contractions from our abdominal sensor compared to a gold-standard tocodynamometer (GE Corometrics 250cx; GE Healthcare). We demonstrate the sensor’s capability of detecting uterine contractions during labor. A flow diagram of the algorithm is shown (*SI Appendix*, Fig. S3*B*). Performance of the abdominal sensor was assessed by comparing peak counts with the gold-standard system. Discernment of individual contractions was made by an obstetrician (J.R.W.).

### Clinical Validation and Deployments in Both High-Resource and Low-Resource Settings.

We present performance, feasibility, and usability of the sensors, both at the individual and population level, in the total cohort of 576 pregnant people. This includes 91 (16%) participants recruited at Prentice Women’s Hospital (Chicago, IL) using all three sensor systems (chest, limb, and abdomen). The sensors were placed on the participant for a period of time that did not interfere with clinical care ranging from 25 min to several hours during both nonstress testing (*n* = 59) as well as active labor (*n* = 32). An additional 485 (84%) pregnant people were recruited in Lusaka, Zambia using the maternal sensors only (chest and limb). Those in the Zambian cohort are participants in the Limiting Adverse Birth Outcomes in Resource-Limited Settings (LABOR) study (clinicaltrials.gov: NCT04102644), which is documenting labor, delivery, and the early postpartum period in 15,000 mother–newborn pairs via detailed physiological data generated by these sensors along with laboratory (blood and urine samples) and clinical data. Here, the sensors were placed on the participants during their entire labor ranging from 1 h to more than 24 h. We note that the participants recruited in the low-resource setting were all actively in labor with imminent delivery. The resulting datasets will be used to generate new predictive algorithms to prospectively stratify patients according to their risk of adverse outcomes and signal actionable intrapartum diagnoses. All participants provided written informed consent with research protocols approved by the Institutional Review Boards of Northwestern University (STU00205895) and the University of North Carolina (19-0765). The final cohort recruited in the pilot study included a wide range of maternal ages (18 to 43 y), races and ethnicities, and gestational ages (25 to 41 wk) (*SI Appendix*, Table S1). In addition, a cohort of pregnant subjects and 10 of their obstetrical providers were surveyed about their experiences with the system (*SI Appendix*, Fig. S4). More than 85% of laboring people surveyed (41 out of *n* = 48 total respondents) indicated a positive or very positive experience with the wireless sensors. Among surveyed providers (*n* = 10), all indicated a positive or very positive experience with the wireless sensors.

### Continuous Surrogates of Systolic BP Derived from PAT and Pulse Transit Time.

**Fig. 4.**
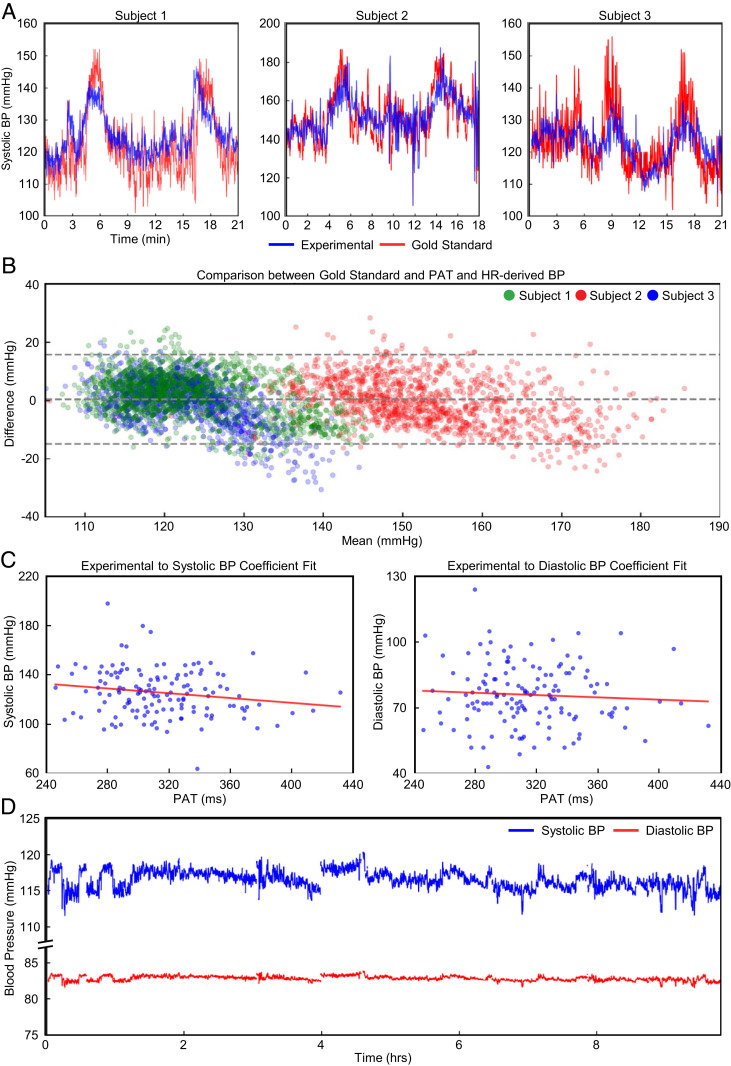
Continuous blood pressure correlation. The derivation of continuous BP from PAT and HR measurements. (*A*) In a cold-pressor test, we use the first 50 s of sampled data to derive linear coefficients for PAT. For each sample, we overlay the gold-standard SBP. (*B*) A Bland–Altman plot comparing the calibrated PAT to the gold-standard SBP from the cold-pressor test is presented. (*C*) An inverse relationship across all participants is derived between PAT and sampled BP cuff measurements. (*D*) *n* = 1 is shown for the converted systolic and diastolic BPs from PAT.

Hypertensive disorders of pregnancy (e.g., gestational hypertension and preeclampsia) cause significant morbidity and mortality in both high-resource and low-resource settings. While BP is ascertained with traditional sphygmomanometers, these systems are noncontinuous. Because they also require clinical staff to correctly deploy each time a measurement is indicated, their actual utility may be limited in low-income settings where clinical personnel resources are limited. Herein, we demonstrate the derivation of a continuous surrogate of systolic BP (SBP) via PAT and HR without the need for clinical staff to initiate, collect, and then record any measurements after an initial calibration. Previously, we have demonstrated the accuracy and performance of this method, which has been improved with the addition of HR (22, 31). In Fig. 4*A* we demonstrate a high degree of agreement with traditional sphygmomanometers, capturing BP at discrete time points with average errors less than 10 mmHg. A Bland–Altman plot in Fig. 4*B* demonstrates the accuracy of our method, which has a mean difference from the sphygmomanometer of 0.4 mmHg (SD 7.8 mmHg). In Fig. 4 *C* and *D*, we demonstrate the ability of our methods to capture measurements over 10 h of labor with our sensors after a single point calibration with a sphygmomanometer. We derive an inverse relationship between PAT and BP measurements across the entire cohort of pregnant women in the low-resource setting. The result is a continuous surrogate measurement of systolic and diastolic BP. However, limitations to this PAT-based BP measurements should be clearly outlined. First, PAT-based measurements still require an initial calibration with a gold-standard BP device (e.g., a traditional oscillometric BP cuff or an arterial line). This calibration step then leads to further errors. Our demonstration here in *n* = 3 healthy normal subjects in highly controlled settings with variable BP achieved via the cold-pressor method shows an average mean and root mean square error of 0.6 and 5.31 mmHg for SBP and 0.2 and 7.07 mmHg for diastolic BP, meeting the tolerable error of 10 mmHg from consensus guidelines (32) for BP measurement systems. Prior work, pending publication in *n* = 23 subjects with in-dwelling arterial lines show an average error of 8.8 mmHg for SBP and 6.5 mmHg for diastolic BP—which also meets consensus guidelines for tolerable error (31). While our data show cold-pressor-induced hypertensive episodes, further work is necessary to fully validate our PAT-based measurements for elevated BP that follow established guidelines for clinical testing with at least *n* = 85 subjects (32) prior to wider-spread clinical use.

### Time-Series Data of Vital Signs during Labor.

**Fig. 5.**
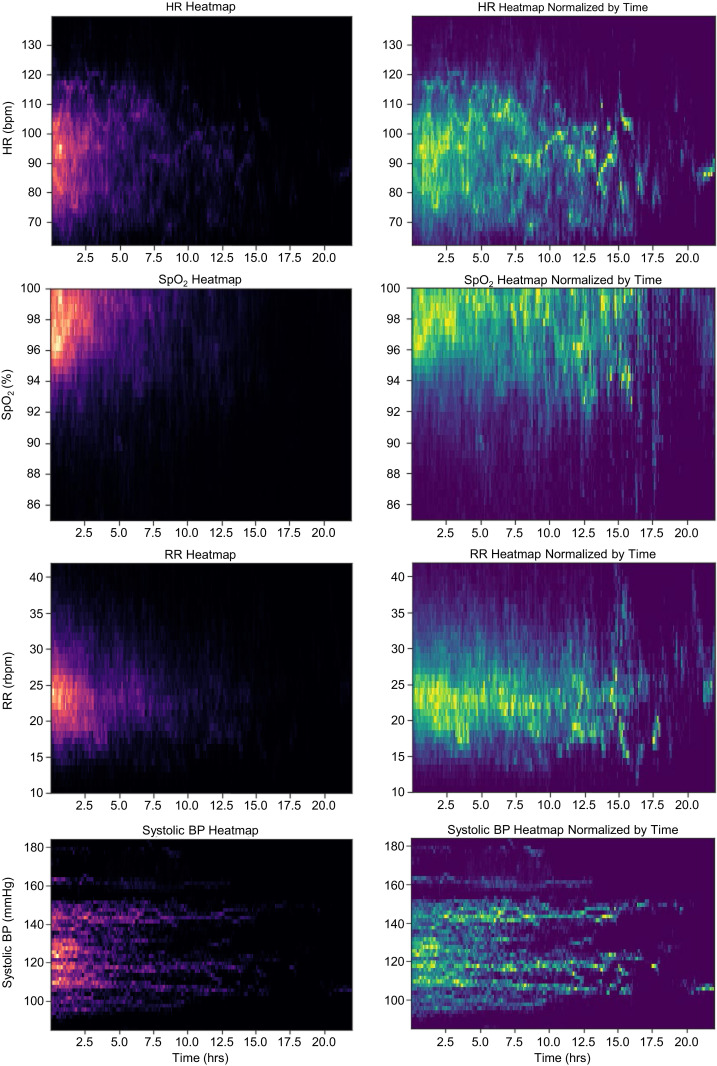
Heat-map analysis of maternal vital signs for laboring women in low-resource settings. A heat map of each vital signal is generated for all 485 participants. (*Left*) Plot (in red–black) for each vital is a heat map without normalization as the majority of labors last less than 5 h. (*Right*) Plot (in yellow–green) is normalized by frequency with a 4-h window to illustrate average vital signs over longer labors.

The ability to continuously track vital signs allows for detailed physiological profiling of a person’s health status during the course of labor. In our cohort of *n* = 485 laboring participants in low-resource setting (Lusaka, Zambia) we demonstrate heat maps illustrating median vital signs for HR, SpO_2_, RR, and PAT converted to BP in Fig. 5. Participant density is depicted through brighter colors as opposed to darker tones, where occurrence is scarce. As the majority of the monitoring period during labor did not extend beyond 5 h, we provide additional graphical plots where each vital sign is normalized every 4 h up to 24 h to illustrate expected vital sign parameters for participants over the course of their active labor. The future utility of this platform allows for automated alerts to qualified healthcare providers to identify participants who deviate from expected values in a time-sensitive manner across the labor continuum for essential maternal vital signs.

### Real-Time Tracking of Maternal Body Position during Labor.

**Fig. 6.**
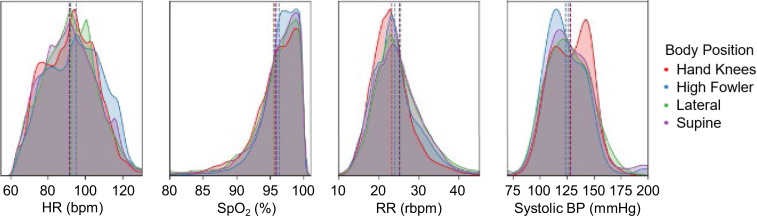
Body position and vital signs. Small, but clear, differences are seen in maternal vital signs based on body position for HR, SpO_2_, RR, and SBP derived from PAT. A vertical line is depicted in each of the vital sign distribution plots to denote the mean value for the specific body position.

In Fig. 6 we demonstrate the response of HR, SpO_2_, RR, and PAT converted to SBP categorized by different laboring body positions (supine, lateral, hands and knees, and high Fowler’s). We automatically categorize these four common laboring positions based on the three-axis IMU outputs. A vertical line is depicted in each of the vital sign distribution plots to denote the mean value for the specific body position. This monitoring system is able to automatically categorize pregnancy body positions with comprehensive vital signs monitoring. This enables the future ability to quantify physiological responses to body position changes to optimize birth outcomes. We present differences in average maternal vital signs in *SI Appendix*, Table S2 based on positional changes.

## Discussion

Current pregnancy monitoring with continuous CTG in high-resource settings has undergone little innovation over the past several decades. These systems require a large central monitor with rigid pucks (7 to 9 cm in diameter) strapped to the maternal abdomen that are wired to a local receiver and monitor (33). In low-resource settings, lower-complexity tools such as partographs, Pinard stethoscopes, and intermittent handheld Doppler US have been used to track fetal well-being and labor progress (14, 34, 35). However, there is concern that these tools lack clear evidence of clinical benefit in low-resource settings (14, 35). While there are newer platforms that assess fetal ECG (17, 36), these still require bulky systems adhered to the body or wired to base units. Use of these newer systems in multiple-gestation or preterm fetuses have not been validated extensively and would be considered off-label if used in these clinical scenarios. Perhaps most notable, of all the wireless pregnancy monitoring devices currently approved for clinical use, none incorporates maternal vital signs. Assessing maternal vital signs such as HR, RR, SpO_2_, and BP requires additional systems wired to the body and configured to another monitoring system that is often not interoperable. Ultimately, with the technologies presently in use, evaluation of the full spectrum of both maternal and fetal health requires a complex and expensive configuration of wired sensors and large, stationary base units.

We present a wireless monitoring platform that provides comprehensive assessment of both the mother and fetus with compatibility across a wide range of mobile devices. The use of Doppler in our abdominal sensors allows the system to potentially monitor nonsingleton pregnancies with multiple patches. Beyond traditional vital signs, this system offers clinically relevant measurement modalities specific to pregnancy monitoring. This system is able to provide continuous body positioning categorization and continuous BP assessments and has been deployed in a significant number of pregnant women. The ability to track body position continuously and concurrently with maternal and fetal vital signs offers an opportunity for improved monitoring and the ability to manage labor by modifying maternal body position as clinically indicated (37). Altering maternal positioning during labor as a means of fetal resuscitation is common (38–40). Some studies suggest upright postures have been associated with shorter early labor stages, a trend toward fewer cesarean deliveries, and less use of analgesia (41). The supine position is often avoided given worse uteroplacental perfusion because of maternal hypotension (42) and prolonged labor (43). Previous research efforts have also demonstrated maternal position impacts maternal blood flow, labor progression, and patient comfort (44–46). Other studies have found that the hands-and-knees position has not been associated with negative labor outcomes (47). Despite these studies, research on the effects and benefits of laboring positions has been largely limited because of inability to monitor and collect data in clinical settings with fidelity. The system we present here provides objective and real-time data outputs on the impact of maternal body position on both maternal and fetal health with the ability to assess how position may be associated with clinical outcomes and the potential to include automated alerts to patients and providers for body position changes if indicated. In previous work, we demonstrate the ability for the chest- and finger-mounted sensors to monitor vital signs in both children and adults; the abdominal sensor allows for maternal–fetal monitoring of pregnant women in both high- and low-resource settings as well as the clinical and home settings (22, 23). Future efforts will evaluate the link of maternal vital signs with fetal physiological parameters in hopes of identifying signs of distress earlier than what is possible today.

In both high- and low-resource settings the burden placed on clinical staff in order to obtain physiologic information on a woman and her fetus is significant. The system provided here can be configured so as to alert providers only when thresholds of vital signs that are indicative of concern are crossed. Additionally, these systems can provide a wealth of data in different clinical settings with labeled clinical outcomes, such as that generated by the LABOR study, that offer the opportunity for more population-specific and sensitive delineation of maternal or fetal status to support clinical decision-making. Furthermore, the sensors are able to be used seamlessly across the entire continuum of maternal, fetal, and neonatal care. Additionally, as demonstrated by our previous work, neonatal vital signs can also be tracked with the same sensors in both high-resource and low-resource settings as well as support kangaroo care (22, 48).

Cost remains a critical barrier particularly in low-resource settings for acquisition and use of perinatal monitoring technologies (49). The use of medical devices in low-income settings often is not sustainable even when donated. The key drivers of this failure are multifactorial and include the cost of maintenance, lack of skilled technicians to maintain the equipment, lack of spare parts, and high consumable costs (50). The sensor systems presented here are designed to address these challenges. First, the sensors themselves are low-cost (estimated <$40 per sensor at large-scale manufacturing) and fully reusable after a wireless recharge and simple cleaning procedure. The reusability of the sensors dramatically lowers the daily cost of patient monitoring. The consumables of the system are also low-cost and consist of only single-use hydrogel adhesives and latex-free fabric strap (estimated <$0.25 per use). Finally, the system leverages a wide range of widely available mobile devices (Android or iOS) to display, store, and transmit data. By 2025, it is estimated that there will be nearly 700 million smartphones in sub-Saharan Africa, making these mobile devices readily available to be used in order to operate the sensors for healthcare applications in even the most rural settings (51).

Medical monitoring is a cornerstone of modern prenatal and peripartum care. Continuous data of maternal BP, HR, SpO_2_, uterine contractility, and FHR are potentially life-saving if acted on, but access is heavily dependent on clinical infrastructure, availability of skilled healthcare providers, and integration of disparate devices and their displays (52). The system we present here offers comprehensive and advanced measurement modalities with evidence of feasibility, performance, and user-preference acceptability among laboring women in both high-resource and low-resource settings for both in-hospital and remote monitoring applications. Ongoing efforts include monitoring up to 15,000 laboring women (LABOR Study; NCT04102644) with the expectation that outputs from these sensors will enable early warnings for clinically meaningful end points such as hemorrhage, emergency cesarean section, fetal demise, and maternal death.

## Materials and Methods

### Sensors.

A four-layer flexible printed circuit board is fabricated for the chest and limb sensors, while the abdominal sensor is made of two layers. The material stack-up consists of sheets of 1oz copper with intermediate layers of polyimide. Integrated circuit components are then assembled onto the boards and an outline is laser-cut to produce the final board. The electronic boards are packaged into a silicone (Silbione RTV 4420; Elkem) layer cast onto an aluminum mold, which is then compressed under regulated temperature and pressure by using a hydraulic press (Carver Press; Carver Inc.).

### Continuous BP Derivation.

We use the detected R-peaks from the ECG signal and pulse peaks from the PPG signal to derive PAT, which represented the time for a pulsatile pressure wave to reach the limb location from the heart. To demonstrate the utility of this measurement, we show in three healthy subjects (*n* = 3) continuous BP derived from our experimental sensor system compared to a bulky gold-standard continuous BP monitoring system (Finapres Nova; Finapres Medical Systems). PAT and HR are sampled and calibrated to the SBP obtained from a gold-standard monitor. In this scenario, we demonstrated the responsiveness of our system to controlled changes of BP with a cold-pressor test, a well-established, safe, and reproducible method to induce a BP response (53).

For each LABOR study participant, we measured BP via a clinically calibrated sphygmomanometer and recorded the time of measurement. These data points are then used to find the global linear coefficient of PAT-to-BP translation for both systolic and diastolic BP. We calculated the difference between the PAT-derived BP and actual measurements and applied the necessary offsets.

### Body Position Derivation.

As the devices were placed in fixed location across the participants, we can use the three-axis accelerometer data to categorize and derive body postures. The baseline value of the accelerometer is sampled at the same rate as other vital signs. With an agglomerated dataset on all subjects with adequate signal quality, we used 50,000 randomly sampled data points and a Gaussian mixture model with *n* = 8 clusters to find the representative accelerometer values. Those eight clusters are further merged and translated into four postures: lateral, high Fowler’s, hands–knees, and supine.

### Time-to-Vital Analysis.

All time-series analyses were done on Python with the scipy package for signal processing and the matplotlib package for graphing. A session time-to-vital distribution heat map is generated for HR, RR, SpO2, and derived SBP from a low-resource setting (*n* = 485). Heat maps located on right column of Fig. 6 show the time-based normalized results to better visualize the vital distribution trend over the session duration.

## Supplementary Material

Supplementary File

Supplementary File

## Data Availability

All study data are included in the article and/or supporting information.
