# Supplementary material for: Comprehensive pregnancy monitoring with a network of wireless, soft, and flexible sensors in high- and low-resource health settings
Source: Proc Natl Acad Sci U S A. 2021 May 10;118(20):e2100466118. doi: 10.1073/pnas.2100466118 (PMC8157941; doi:10.1073/pnas.2100466118)
Supplement: Supplementary File [file pnas.2100466118.sapp.pdf]

PNAS
www.pnas.org

Supplementary Information for

Comprehensive Pregnancy Monitoring with a Network of Wireless, Soft and Flexible Sensors in High and Low Resource Health Settings

Dennis Ryu, Dong Hyun Kim, Joan T. Price, Jong Yoon Lee, Ha Uk Chung, Emily Allen, Jessica R. Walter, Hyoyoung Jeong, Jingyue Cao, Elena Kulikova, Hajar Abu-Zayed, Rachel Lee, Knute L. Martell, Michael Zhang, Brianna R. Kampmeier, Marc Hill, JooHee Lee, Edward Kim, Yerim Park, Hokyung Jang, Hany Arafa, Claire Liu, Maureen Chisembele, Bellington Vwalika, Ntazana Sindano, Bridget M. Spelke, Amy S. Paller, Ashish Premkumar, William A. Grobman, Jeffrey S. A. Stringer, John A. Rogers, Shuai Xu

This PDF file includes:

Supplemental Text
Tables 1 to 2
Supplemental Figures S1 to S5
Supplemental Audio

Supplementary Information Text

Wireless Maternal-Fetal Sensor System Design

The chest sensor's biopotential AFE offers features including EMI filtering and DC lead-off detection. The sensor is compliant with the IEC 60601-2-47:2012 standard, and measures clinical-grade ECG signals with an average noise of 3.1 μV_{P-P} and a common-mode rejection ratio (CMRR) greater than 100 dB. Additional features include a bioimpedance channel and pacemaker detection. The 6-axis IMU has an acceleration range from ±2 to ±16 g and an angular rate range between ±125 to ±2000 dps, while having an accuracy of ±40 mg and ±3 dps respectively. The clinical-grade thermometer offers a measurement accuracy of 0.1 °C and can measure between 0 to 50 °C. The Bluetooth Low Energy (BLE) system-on-a-chip (SoC) controls the biopotential AFE and IMU through a Serial Peripheral Interface (SPI) communication protocol and the thermometer through the Inter-integrated Circuit (I^2C) communication protocol.

The acquired signal from the optical AFE in the limb sensor is processed through a high-resolution 19-Bit delta-sigma ADC with an integration time between 14.8 and 117.3 µs. The AFE offers additional configurations including ambient light cancellation, input capacitance adjustment, and picket fence detection, which help eliminate artifacts in the signal. The same clinical-grade thermometer measures skin temperature with a measurement accuracy of 0.1 °C and can measure between 0 to 50 °C. The BLE SoC controls the pulse oximetry module through the SPI communication protocol and the thermometer through the I^2C communication protocol.

The two 30 mm diameter half-moon piezoelectric transducers (Xinhua YCB Ceramics and Electronics Co., Ltd.) in the abdominal sensor have a resonance impedance less than 1 Ω and a static capacitance between 2.4 to 2.8 nF, and are tuned to resonate at 3 MHz and are incorporated as the transmitter and receiver for the Doppler effect. Through analog feedback and cascaded filtering, the signal is fed into the BLE SoC. which is designed to detect low-power signal sources down to a noise of 1 μV_{P-P} and a CMRR of 110 dB. The AFE offers a programmable gain up to 24, a 24-Bit delta-sigma ADC, and an internal reference and onboard oscillator. The BLE SoC controls the biopotential unit through the SPI protocol.

Real-Time Measurement of Clinical Data for Maternal Health

Calculation of SpO_2 involves a Butterworth bandpass filter (f_{c1} = 0.8 Hz, f_{c2} = 5 Hz) and adaptive threshold decaying peak detection. Using the infrared channel for primary peak detection, peak information such as peak height and valley height is cross-referenced between the channels and stored into a buffer. First, we performed heuristics to ensure the validity of detected peaks. Given the assumption motion artifact is often local, the buffer is segmented into smaller time frames and heuristics calculation is repeated in a breadth search manner. Combined with statistical metrics and the depth, we derive signal quality that is effective in determining the presence of motion artifact and non-pulsatile signal. Finally, with the peak information in a valid segment of the buffer, we derive the SpO_2 value through a ratio of pulse amplitude between the red and infrared channels

For the calculation of RR, the x- and y- channels of the accelerometer are bandpass filtered (f_{c1} = 0.1 Hz, f_{c2} = 0.8 Hz). We linearly interpolate R-peak amplitudes detected from the ECG signal and feed into the bandpass filter to derive ECG-based respiration flow. Each of these channels is passed onto peak detectors and a series of heuristics to determine signal qualities similar to the method used for SpO_2 derivation. For each second, the channel with best signal quality above a certain threshold is dynamically selected to derive the respiration rate.

Fetal Heart Rate and Uterine Contraction via a Soft, Flexible, and Wireless Abdominal Sensor

The acoustic signal from the abdominal sensor penetrates soft tissue and is reflected by abrupt changes in acoustic impedance. Upon reflection, the waves are altered to a different frequency with the Doppler effect. The waves are then demodulated to the appropriate frequency for further signal processing.

The audible fetal phonocardiogram from the Doppler US is high pass filtered (f_c = 1 Hz) through a Hilbert transform algorithm and scaled to obtain a positive envelope function. The envelope function is segmented and processed with an autocorrelation function to identify peaks of recurring frequencies. The intervals of these frequencies are continuously updated and used to calculate FHR.

The dual EHG signal obtained by the two channels in the abdominal sensor is bandpass filtered (2nd order f_{c1} = 0.3 Hz, f_{c2} = 1 Hz) to produce a signal corresponding to EMG. A moving Hanning window of 30 seconds then samples the data and yields an envelope signal. The envelope signals of the two channels are aggregated into a single waveform and uterine contraction is obtained.

Materials and Methods

Sensors

Soft silicone elastomer encasings are prepared as an enclosure for the chest and limb boards. Silbione RTV 4420 (Elkem) mixed with a bio-compatible dye are cast into an aluminum mold. A hydraulic press (Carver) compresses the mold with a pressure of 1000 lbs at 220°F for 3 minutes, yielding a fully cured layer with a thickness ranging in between 200-300 µm. Two separate layers, top and bottom, are manufactured for the assembly of one sensor. For the chest sensor, a pre-cut 96042 (3M) Double Coated Silicone Tape is placed onto the bottom layer for improved adhesion to the deposited electrode of the printed circuit board. In the process, the sub-islands are folded for optimal distribution of IC components, thereby reducing the board's two-dimensional surface area by up to 250%. Pre-compressed serpentines on the board provide increased stretchability once the sensor is fully manufactured. A mixed Ecoflex 00-30 (Smooth-On) compound is poured into the top layer for added conformability, and the two halves are placed back into the press for fusion. After full encapsulation, the sensor is outlined using a die cut and a unique identifier is engraved using a carbon dioxide laser.

The construction of the abdominal sensor is depicted in greater detail (SI Appendix, Fig. S5). In Fig. S5A, we show that the polyimide material and copper traces that make up the flexible printed circuit board are first manufactured, and then the necessary electronic IC components are placed on top. From the completed circuit, Fig. S5B and S5C demonstrate how the exterior planes are folded to reduce the two-dimensional surface area by approximately 24%. A photopolymerized resin mold is 3D-printed to house the ultrasonic transducers, which is attached to the underside of the board. The 30mm-diameter transducers are secured with a cyanoacrylate adhesive. The board, fully assembled and folded, is ready for encapsulation. Like the chest and limb sensors, a silicone elastomer is cast into an aluminum mold but is spin coated at 250 rpm instead. This procedure is done for both the top and bottom layers, and they are cured in an oven at 100°C for 20 minutes. The cured bottom layer is laser-cut to expose the ultrasonic transducer and the layers are assembled with the abdominal board inside. Fig. S5D shows an expanded diagram of the finished abdominal sensor, which shows all layers including the top silicone layer, IC components, flexible printed circuit board, transducer housing, and the bottom silicone layer.

Continuous Blood Pressure Derivation

The theory behind and accurate determination of BP from PAT derived from this sensor system has been reported previously compared against indwelling arterial lines and advanced modeling (22, 23, 25). The validation of PAT-based BP measurements has been extensively performed previously in multiple publications and on more than 70 different participants collectively, ranging from newborn to adults (22, 31). This demonstrates the wide deployment of this technology and its applicability in multiple age groups.

In the cold pressor test, the left hand is immersed in ice water for 1-2 minutes two separate times, spaced 10 minutes apart, while the right hand has the limb sensor adhered to the participant. For this study, we sampled PAT at 3 Hz for near beat-to-beat values and later performed outlier removal and moving average. Both PAT and HR are collected for the first fifty seconds for each subject, which are then calibrated by ordinary least square regression to the SBP obtained from either a traditional BP cuff or a desktop continuous BP monitor using the linear relationship BP = a(PAT) + b(HR) + c. BP is then continuously calculated from this point forward with the experimental system compared to the gold standard.

Table 1. Demographic characteristics of maternal patients admitted in the maternity center in this study

	**High-Resource Setting Chicago, IL**	**Low-Resource Setting Lusaka, Zambia**
**Subjects**	91	485
**Median Age (range)**	31 (18-43)	27 (18-49)
**Care Setting**		
Active Labor	32	485
Non-stress Testing	59	0
**Gestational Age**		
Preterm	31	211
Early Term	13	198
Full Term	41	72
Late Term	3	4
Post Term	0	0
Not Reported	1	0
**Race**		
Black	26	485
Asian	5	0
Latino	12	0
White	39	0
Other	1	0
Not Reported	4	0

Table 1. Early term: 37 weeks, 0 days and 38 weeks, 6 days. Full term: 39 weeks, 0 days and 40 weeks, 6 days. Late term: 41 weeks, 0 days and 41 weeks, 6 days. Post term: 42 weeks, 0 days or later.

Table 2. Vital Signs Corresponding to Maternal Positioning During Labor

**Mean** Posture	Mean HR (bpm) ± SD	Mean SpO_2 (%) ± SD	Mean RR (rbpm) ± SD	Mean SBP (mmHg) ± SD	Mean DBP (mmHg) ± SD	Mean PAT (ms) ± SD	Mean Chest Temp (°C) ± SD	Mean Limb Temp (°C) ± SD
Hands and Knees	92 ± 14	96 ± 4	23 ± 5	129 ± 18	79 ± 14	315 ± 32	35.8 ± 0.9	34.5 ± 2.0
High Fowler's	95 ± 15	97 ± 3	24 ± 6	124 ± 19	76 ± 14	311 ± 29	35.4 ± 0.9	34.1 ± 2.4
Lateral	92 ± 14	96 ± 4	26 ± 7	126 ± 20	76 ± 14	312 ± 31	35.7 ± 0.9	34.3 ± 1.9
Supine	92 ± 15	96 ± 4	25 ± 7	128 ± 21	77 ± 15	312 ± 28	36.0 ± 1.0	34.2 ± 2.0
Overall	92 ± 15	96 ± 4	25 ± 6	127 ± 20	77 ± 14	312 ± 30	35.6 ± 0.9	34.3 ± 2.1

Table 2. Mean and 95% Confidence Interval for RR, HR, PAT (BP), and SpO_2. For each vital sign we generate a statistical specification on different postures for all participants in the low-resource setting. Vitals are calculated to the nearest first decimal place for improved 95% percentile distinguishability.

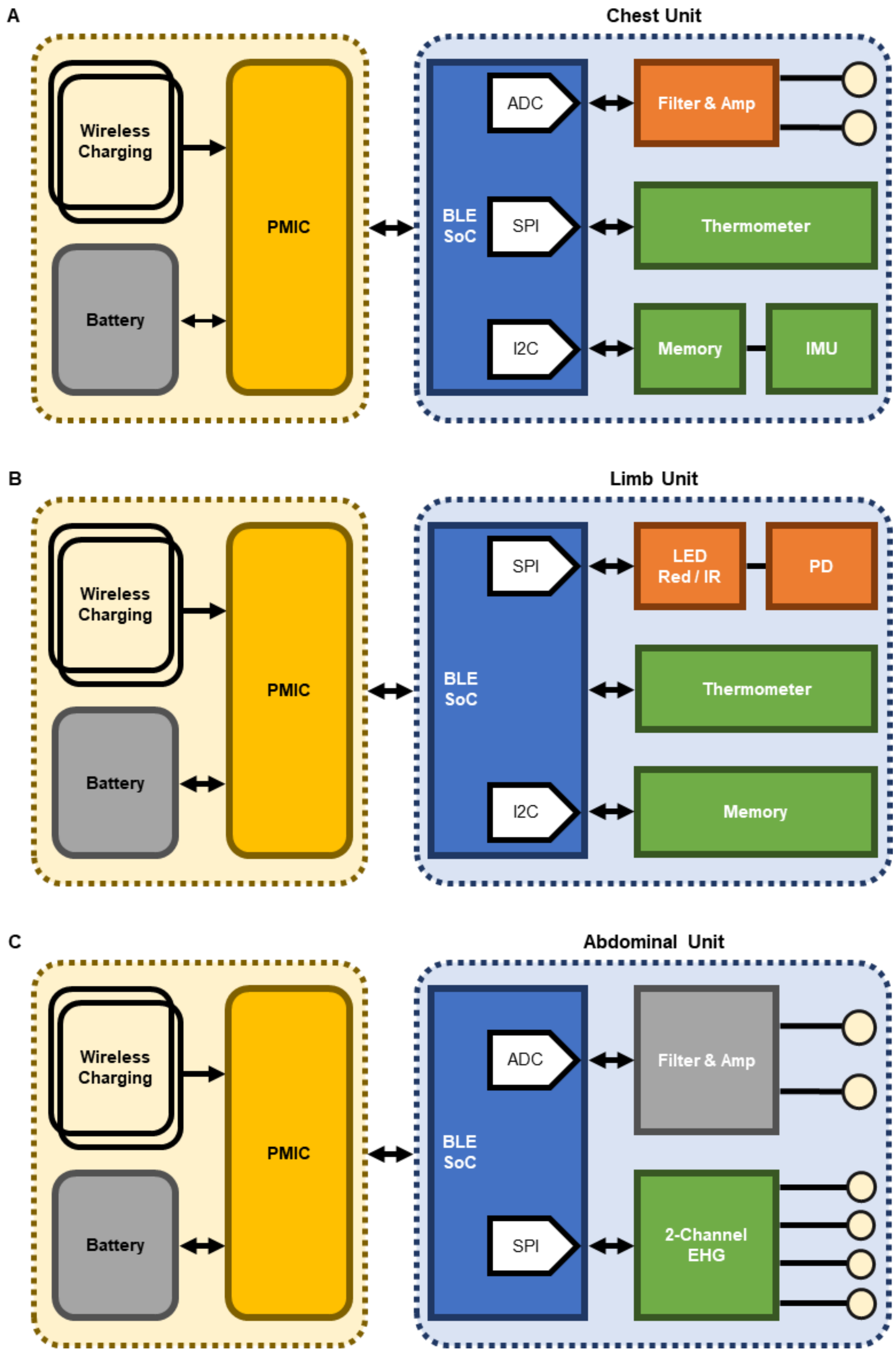

Supplementary Figure S1. Block diagrams of the chest (a), limb (b), and abdominal (c) sensor.
Block diagram for each of the Chest, Limb, and Abdominals sensors shows the modular power, communication, and data schemes.

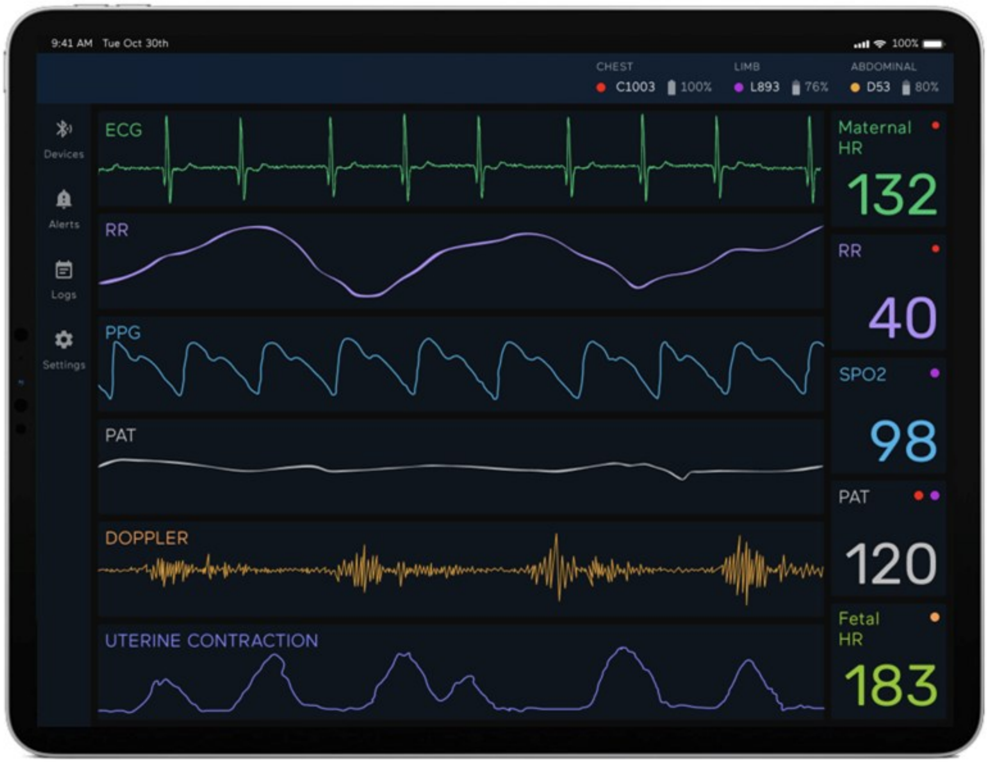

Supplementary Figure S2. A simple platform for remote monitoring of maternal vital signs. An intuitive user interface of the system deployed on a mobile device (iPad).

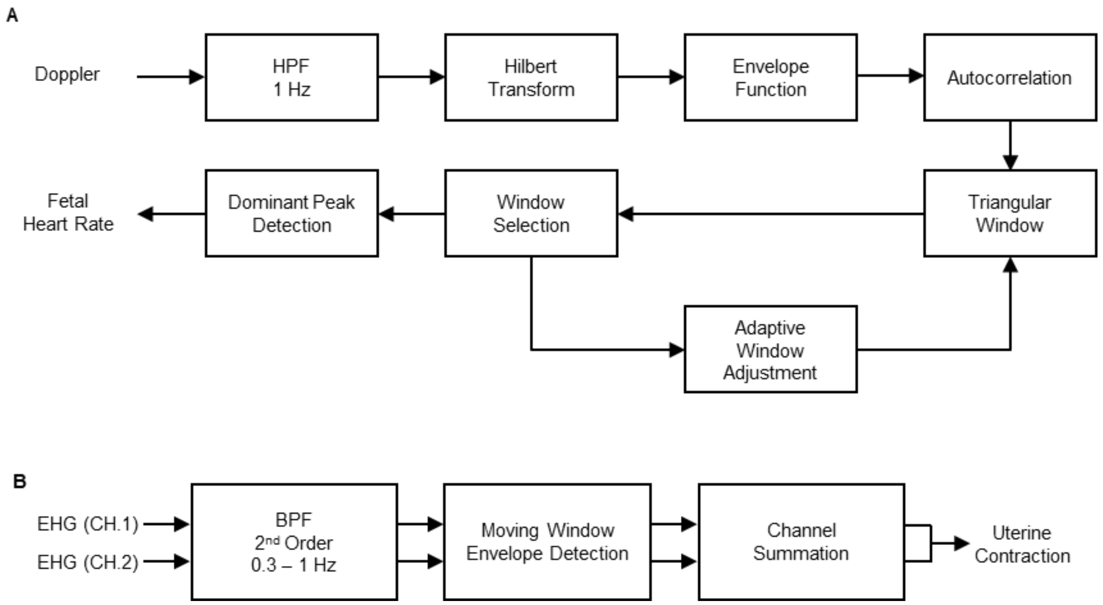

Supplementary Figure S3. Block diagram of algorithms for the abdominal sensor.
Block diagrams of the algorithms for derivation of fetal heart rate and maternal uterine contraction are depicted.

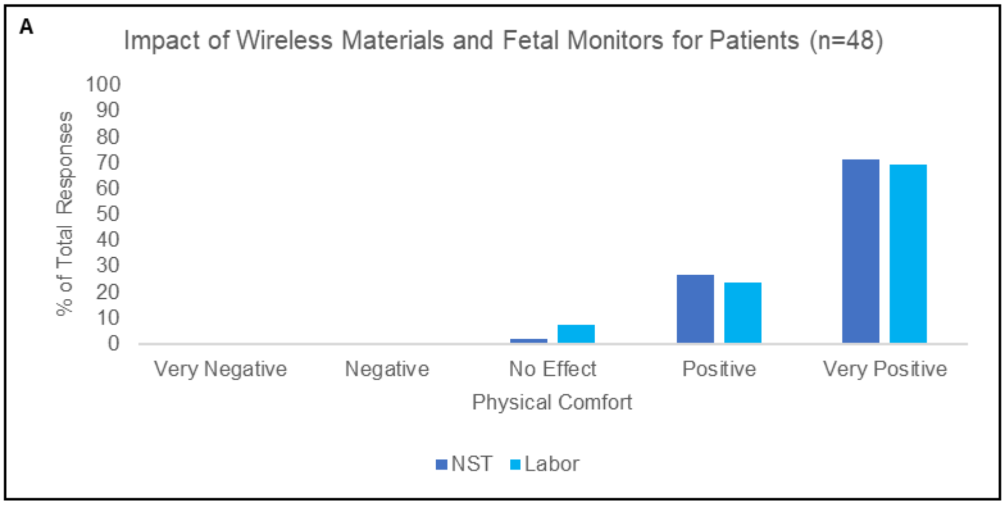

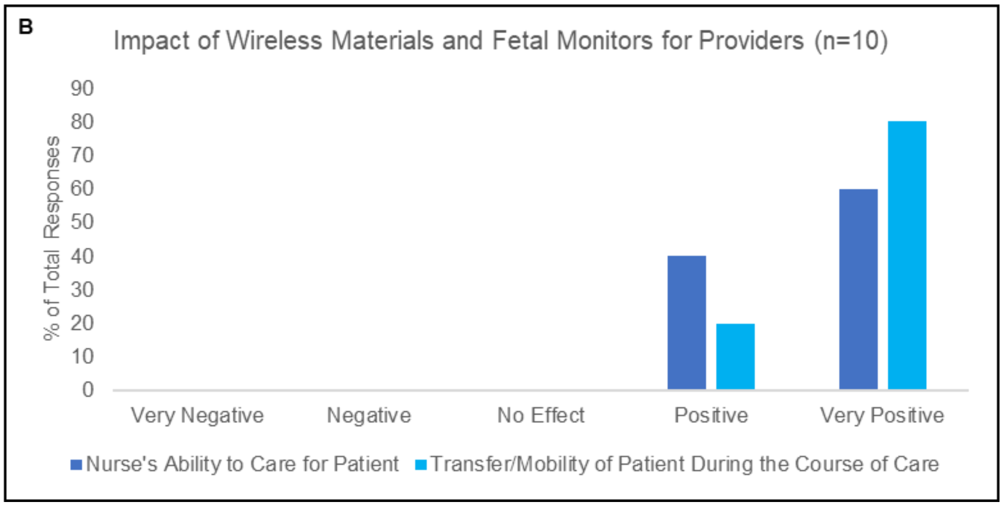

Supplementary Figure S4. Feedback provided by a subset of pregnant women (A) and obstetrical nurses (B).
Usability and user experience feedback was obtained from both the patients and the sensor administrators.

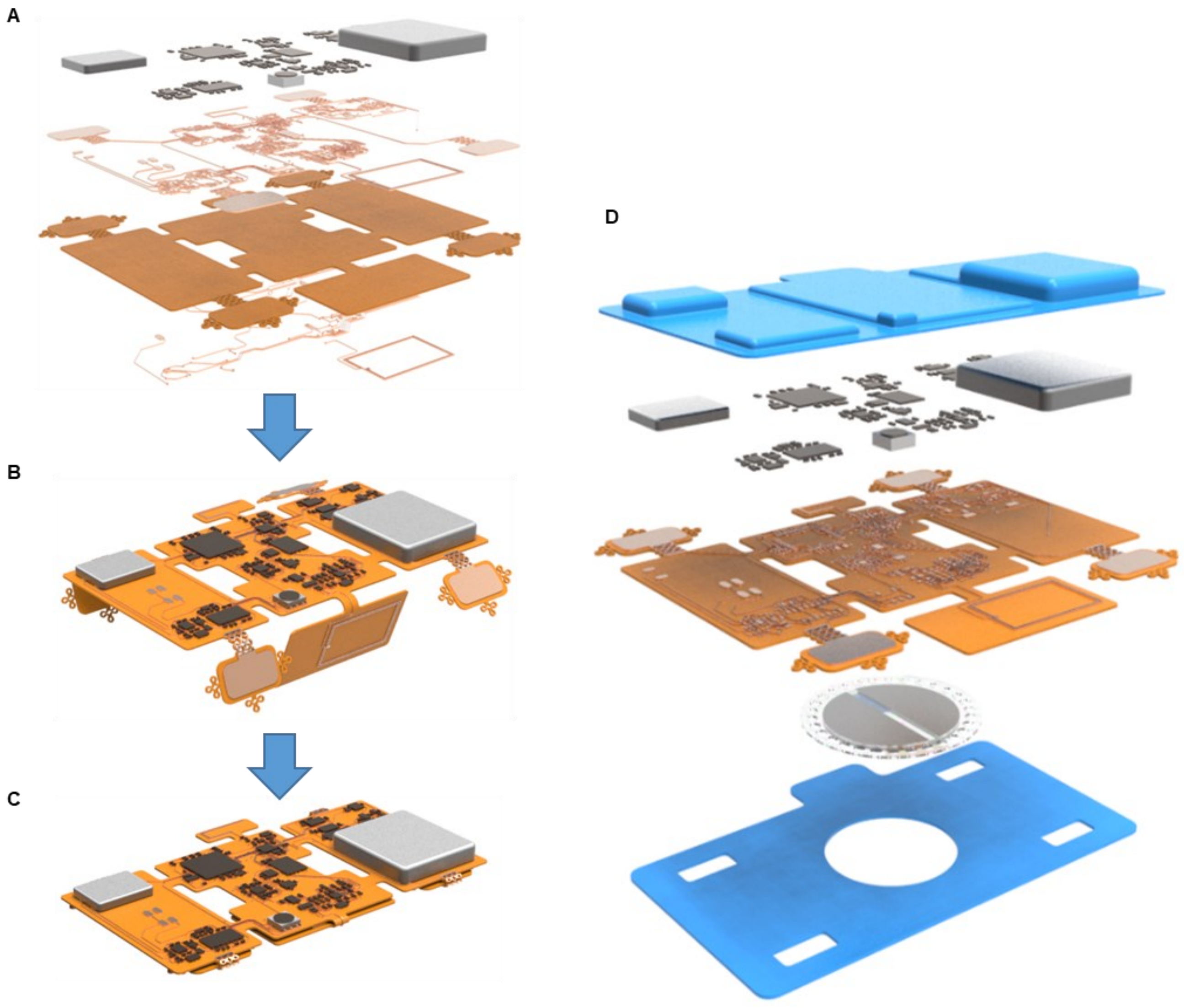

Supplementary Figure S5. The construction illustration of the abdominal unit.
A flexible printed circuit board is fabricated and assembled with integrated circuit components. The sub-islands are folded and encapsulated in a thin silicone layer.

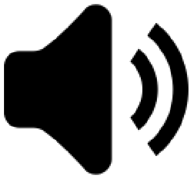

Supplementary Audio. Fetal doppler signal.
A fetal doppler waveform converted to an audible sound discernable by clinical personnel.
